# Supplementary material for: A 3D ray traced biological neural network learning model
Source: Nat Commun. 2024 Jun 1;15:4693. doi: 10.1038/s41467-024-48747-7 (PMC11525811; doi:10.1038/s41467-024-48747-7)
Supplement: Supplementary file 1 — Supplementary Information [file 41467_2024_48747_MOESM1_ESM.pdf]

Supplementary Material for A 3D Ray Traced Biological Neural Network Learning Model

Brosnan Yuen[1], Xiaodai Dong[1*], Tao Lu[1*]

[1]Department of Electrical and Computer Engineering, University of Victoria, 3800 Finnerty Road, Victoria, V8P 5C2, British Columbia, Canada.

*Corresponding author(s). E-mail(s): xdong@ece.uvic.ca; taolu@uvic.ca;

S.1 Background on central nervous system

Unlike artificial neural networks, the human central nervous system (CNS) [S1] dynamically adapts to changing sensory input and evolving objectives. The CNS can compensate for neuron loss due to aging, infections, and injuries by rearranging the neural structure. Moreover, it can create new neurons to increase memory capacity and to learn new skills. Glial cells and neuron cells make up the majority of the CNS, where there are approximately 100 billion glial cells and 100 billion neurons [S2].

Neurons play a huge role in cognitive functions as they are responsible for processing stimulus from sensory input. There are two primary modes of signalling between neurons: electrical signalling and chemical signalling [S3]. The electrical signals are shaped like Gaussian pulses, of which the peak voltage and the pulse width are fixed. Information is encoded in the frequency and the timings of the pulses. Mild stimulus produces low-frequency pulses, while intense stimulus produces high-frequency pulses. Electrical pulses travel much faster than chemical signals, and thus electrical signals are used for fast responses like motor functions. However, electrical signals have very low bandwidth, as they can only operate on a single channel between two neurons. On the other hand, chemical signals like neurotransmitters have a very high bandwidth. Over 200 different neurotransmitters have been discovered [S4] and each neurotransmitter is semi-independent of the other neurotransmitters. This is similar to having 200 independent communication channels between two neurons. Furthermore, electrical pulses can trigger chemical signals and chemical signals can trigger electrical pulses, which leads to many different combinations of interactions.

Neurons have a very complex structure consisting of axons, axon branches, axon terminals, synapses, dendrites, and soma [S5]. Each neuron has only one axon that ranges from 100 μm to $80,000$ μm [S6]. However, each axon can split into multiple axon branches, where each axon branch connects two or more neurons. They are also responsible for carrying electrical pulses and neurotransmitters between the neurons. To ensure the transmitted signals do not degrade, the insides of the axons are made out of a low-impedance conductor, while the outsides are wrapped by high-impedance glial cells. On average, there are around 1 to 11 axon branches per neuron [S7], which means each neuron is connected to somewhere between 1 and 11 other neurons. When forming new neural connections, the axon begins by walking in random directions [S8]. Subsequently, the axon might grow branches to search for other neurons in parallel. Glial cells and debris might block the axon. Therefore, the axon has to maneuver around glial cells and debris to reach other neurons. Every neuron releases trace amounts of neurotransmitters to its local area. Upon detecting the local presence of neurotransmitters, the axon branch will follow the traces and connect to the excreting neuron, forming a new neural connection. The probability of a new connection forming depends on the shortest unblocked path between two neurons and the neuron growth factor. Shorter paths between two neurons increase the probability of new connections, while longer paths between neurons decrease the probability of new connections. In early childhood, the neuron growth factor is very high so many new neural connections form. In adulthood, the neuron growth factor is low, and unused axons/axon branches are retracted or eliminated by glial cells. However, new neural connections continuously form throughout the CNS's lifespan.

At the end of each axon branch, multiple axon terminals connect to multiple dendrites. Each dendrite can have from 1,000 to 10,000 postsynaptic receptors [S9] with each receptor pertaining to a unique neurotransmitter. All dendrites connect to the soma, which is the cell body of the neuron. Functionally, the soma acts as a capacitor that stores the net charge from every postsynaptic receptor [S10] and it has a diameter ranging from 10 μm to 50 μm [S7]. Assuming the extracellular fluid that surrounds all cells in the CNS has a voltage of 0 mV, the soma's voltage is measured relative to the extracellular fluid. Each soma starts with a resting potential of -70 mV. If a neurotransmitter excites the neuron, then the net positive charge and the voltage of the soma increases. If a neurotransmitter inhibits the neuron, then the net positive charge and the voltage of the soma decreases. Every neuron has a unique threshold voltage that controls the firing of that specific neuron. If the voltage of the soma exceeds the threshold voltage, then the neuron fires a 40 mV electrical pulse into the axon. Afterwards, the voltage of the soma drops down to the resting potential.

Glial cells [S11] perform a variety of functions such as guiding newborn neurons into their specific location, supporting and holding neurons in their place, insulating neurons from each other and the extracellular fluid, synchronizing groups of neurons, controlling the abundance of neurotransmitters, repairing neurons, and removing dead neurons. When neurons are born, they have to migrate to their specific region of the CNS. The migration process is guided by glial cells and neurotransmitters [S12]. However, neurons may get misled by other random factors such as infection and debris.

Newborn neurons begin by attaching themselves to radial glial cells and travel alongside glial cells until the neurons reach their approximate destination. Stuck neurons might stay attached to radial glial cells, while other neurons might detach from radial glial cells partway to follow traces of neurotransmitters emitted from older neurons. The rest of the neurons might get lost or die in the process.

As the CNS ages, radial glial cells specialize into astrocytes, oligodendrocytes, microglia, and other glial cells [S11]. Astrocytes hold the neurons in their place and connect them to the blood supply, while simultaneously controlling the concentration of neurotransmitters by breaking them down in the extracellular fluid. Furthermore, astrocytes can synchronize groups of neuron firings by inhibiting individual neurons. Another type of glial cell, the oligodendrocyte, has the unique function of insulating the axon of neurons in the process called myelination [S13]. This is done to prevent the neurotransmitters and electric charges from leaking out into the extracellular fluid. All neurons start without myelination. However, as time progresses, more and more neurons are myelinated to improve the signal integrity and power efficiency of the CNS.

All glial cells can undergo cell division throughout the organism's lifespan to replace damaged or dead cells [S11]. On the other hand, most neurons can not undergo cell division after adulthood, so they are irreplaceable in the event of a neuron death. As a result, the total number of neurons and neuron connections always decreases after adulthood, in contrast to a stable total number of glial cells.

S.2 Feature comparison of related models

Some transfer learning methods keep the neural network constant across various datasets, while other methods significantly modify the neural network structure to adapt to new problems. The adaptations can be used to overcome changes in the input dimensions or changes in the problem's complexity level. Neural network architectures can be tuned by hand, but they are tedious and slow. However, neural evolution and automatic machine learning (AutoML) can automatically tune and optimize neural networks for new datasets, allowing for faster transfer learning applications. In Ref. [S17], researchers developed a recurrent neural network (RNN) for neural architecture search and transfer learning. To achieve the best result for a specific task, its task embedding is fed into the RNN. Afterwards, the RNN predicts the optimal neural network embedding that contains hyperparameters such as the number of hidden layers, the sizes of CNN kernels, dropout rates, and the types of activation functions. For solving similar tasks, transfer learning is employed by feeding the closest pre-trained task embedding into the RNN and reloading the RNN's state to that task's solved state. That way, the RNN can learn from previously solved tasks to get the best performance for the current task. In the end, the RNN controller reduced training time and computational resources by utilizing transfer learning.

In Ref. [S18], researchers used knee-guided evolutionary algorithm (KGEA) [S23] and evolutionary network pruning for evolving and transferring CNNs. Starting with a neural network template such as VGG-16 [S24], they transferred some of the layers of VGG-16 to a new network using genotypes and neuron pruning. The genotypes encode the number of CNN layers, the number of dense layers, and the dropout rate.

Supplementary Table 1 *Feature Comparison of Related Models*

Model	Transfer Learning	Hyper-parameter Tuning	Neural Pruning	Sparse Matrices	Physical 3D Neuron Positions	Raytraced Neural Connections	Unique Activation Functions
HDNN-TL [S14]	✔	✗	✗	✗	✗	✗	✗
Sparse Convolutional Neural Networks [S15]	✗	✗	✔	✔	✗	✗	✗
Training Sparse Neural Networks [S16]	✗	✗	✔	✔	✗	✗	✗
Transfer Learning with Neural AutoML [S17]	✔	✔	✔	✗	✗	✗	✗
EvoNAS-TL [S18]	✔	✔	✔	✗	✗	✗	✗
SaMuNet [S19]	✗	✔	✔	✗	✗	✗	✗
NeuCube [S20]	✗	✔	✔	✗	✔	✗	✗
HyperNEAT [S21]	✔	✔	✔	✗	✔	✗	✗
DES-HyperNEAT [S22]	✔	✔	✔	✗	✔	✗	✗
**RayBNN**	✔	✔	✔	✔	✔	✔	✔

Moreover, the genotypes dictate which layers are discarded, fixed (not trained), and fine-tuned (trained) in the new neural network. In the first stage, KGEA executes a neural architecture search to find the optimal network by continuously evolving the genotypes and selecting the best genotypes with the highest performance-to-network size ratio. For the second stage, neural pruning is activated to disable individual CNN filters and individual neurons in the dense layers. By utilizing transfer learning in neural evolution, it saves training time because the networks are not trained from scratch.

Aside from transfer learning, there are many research articles focusing on neural evolution/AutoML alone. Xue, et al. [S19] created a novel genetic algorithm for evolving CNNs that makes use of candidate offspring generation strategies (COGS). Firstly, a population of random genotypes is created, where each genotype encodes the building blocks of a CNN in sequential order. Each CNN block contains different CNN filters, connection skips, and dropout rates. Secondly, the fittest individuals are bred together, and the mutation operations modify the offspring's genotypes. Afterwards, the best offspring are selected for the next population and the process repeats itself. This evolutionary pressure pushes the population towards the optimal CNN. Thirdly, COGS is used to find the best mutation operations by analyzing which operations produce the fittest individuals. Similar to the above, the best mutation operations generate the next population of mutation operations.

Real biological neural networks naturally make use of transfer learning to survive changing environments. For example, severed starfish can regrow their limbs and regain control over them, even though many neurons and supporting cells were removed in the process. A person born without any arms can manipulate robotic arms to pick up objects, even though they have never operated an arm before [S25, S26]. There are many classes of bio-inspired artificial neural networks that strive to mimic biological systems. One such class is the spiking neural networks (SNNs) [S27], of which achieves the same performance with less computational resources and better power efficiency. SNNs use fixed amplitude Gaussian pulses to communicate between neurons, where the information is encoded in the delay between pulses or the frequency of the pulse train. Moreover, the numerous pulse trains are not guaranteed to reach the receiving neuron at the same time. As a result, the receiving neuron has to store past information in its memory for it to fire at the correct time. Training SNNs requires highly granular time simulations and adjusting the threshold voltages of neurons. In particular, researchers in [S20] created a 3D SNN by arranging neurons in an ordered cube lattice. Short-distance neural connections are generated based on the small-world radius, where every neuron within the radius is sparsely connected as a small-world network [S28]. On the other hand, long-distance connections (LDC) are randomly assigned based on the LDC probability. NeuCube has many applications in precisely modelling EEGs and functional magnetic resonance imaging (fMRI) of human subjects. For the hardware side, SNNs are widely synthesized into application-specific integrated circuits and state-of-the-art neuromorphic hardware. Ref. [S29], showcases a 6-layer spiking CNN with bounded reified linear unit activation function for CIFAR-10 image classification. The design is physically fabricated onto 2 memristive layers, where each layer has a 5×5 grid of memristive cells. Each cell encodes the weight value as the resistance, and they are accessed using bit lines and word lines. The M-SNN achieves 86% training accuracy with 25 μW total power consumption and 200 μs latency.

Compositional pattern-producing networks (CPPNs) [S30, S31] are a class of neural networks that use neuron positions to determine the neural connections and their respective weight values. For example, we want to find the weight between neuron 0 and neuron 1. To do this, we feed neuron 0's position vector $[x_0, y_0, z_0]$ and neuron 1's position vector $[x_1, y_1, z_1]$ into the CPPN. The input positions go through a series of computations based on the CPPN's phenotypes and output the weight value between those neurons. Afterwards, the process is repeated for all combinations of two neurons to get the weight matrices of the neural network. HyperNEAT [S21, S22, S32] algorithms go further by evolving the CPPNs to the optimal configuration using genetic algorithms. When the CPPN changes, its corresponding network structure and the weights change alongside it.

S.3 Implementation details of the proposed biological neural networks

S.3.A CSR matrices

CSR matrices are used to store the weighted adjacency matrix $\mathbf{W}$ because they are space efficient for sparse matrices and are fast for matrix multiplication. Typically, a CSR matrix consists of a nonzero value vector $\vec{W}_{value} \in \mathbb{R}^{N_v \times 1}$, a row index vector $\vec{W}_{row} \in \mathbb{N}_0^{(N+1)\times 1}$, and a column index vector $\vec{W}_{col} \in \mathbb{N}_0^{N_v \times 1}$.

$$\vec{W}_{value} = \begin{bmatrix} \nu_0 \\ \nu_1 \\ \vdots \\ \nu_{N_v-1} \end{bmatrix}, \vec{W}_{row} = \begin{bmatrix} r_0 \\ r_1 \\ \vdots \\ r_N \end{bmatrix}, \vec{W}_{col} = \begin{bmatrix} c_0 \\ c_1 \\ \vdots \\ c_{N_v-1} \end{bmatrix} \tag{S1}$$

The row index vector $\vec{W}_{row}$ gives the start index r_i and the end index r_{i+1} of a row i that corresponds to a segment on the nonzero value vector $\vec{W}_{value}(r_i : r_{i+1} - 1)$ and on the column index vector $\vec{W}_{col}(r_i : r_{i+1} - 1)$. Moreover, the nonzero value vector $\vec{W}_{value}$ stores the values of the nonzero elements, where N_v is the number of nonzero elements. The column index vector $\vec{W}_{col}$ stores the column indexes of the nonzero value vector. While CSR matrices are used for computing forward pass and backward pass, we use COOrdinate format (COO) matrices to add and delete new weights/neural connections.

S.3.B COO matrices

COO matrices are much more efficient than CSR for adding and deleting weights/neural connections because individual weights/neural connections can be manipulated more easily. The main feature in COO matrices is that row indices, column indices, and value vectors have the same dimensions.

$$\vec{W}_{value} = \begin{bmatrix} \nu_0 \\ \nu_1 \\ \vdots \\ \nu_{N_v-1} \end{bmatrix}, \vec{W}_{row} = \begin{bmatrix} r_0 \\ r_1 \\ \vdots \\ r_{N_v-1} \end{bmatrix}, \vec{W}_{col} = \begin{bmatrix} c_0 \\ c_1 \\ \vdots \\ c_{N_v-1} \end{bmatrix} \tag{S2}$$

For example, if ν_5 representing a neural connection needs to be deleted, then ν_5 is removed from $\vec{W}_{value}$, r_5 is removed from $\vec{W}_{row}$, and c_5 is removed from $\vec{W}_{col}$. In another example, if ν_9 representing a brand-new weight needs to be added, then ν_9 is inserted into $\vec{W}_{value}$ after ν_8, r_9 is inserted into $\vec{W}_{row}$ after r_8, and c_9 is inserted into $\vec{W}_{col}$ after c_8. If multiple weights/neural connections need to be added, then they are appended to the end and the corresponding vectors are sorted to ensure they have the correct order from ν_0,ν_1,ν_2 to ν_{N_v-1}.

S.4 Transfer learning network evolution animation

We created an animation [S33] detailing the transfer learning evolution of the RayBNN on the Alcala dataset, where every λ represents a different training dataset. On the top left, we plot the 3D positions of the cells and the connections between neurons. On the top right, each neuron's unique UAF is plotted as a function of input. On the bottom left, we show the weighted adjacency matrix that represents the weights of the neural connections between the neurons. The sparse matrix has bigger dimensions than needed to account for the growth of the neural network. Having a larger sparse matrix does not hurt the performance as the zero elements are not saved or used in the computations. Additionally, we also reserve some space for the input neurons, so there is a space between the two blocks of matrices. On the bottom right, the weighted adjacency graph displays the degree of separation between the neurons.

Supplementary References

[S1] Cooke, S.F., Bliss, T.V.: Plasticity in the human central nervous system. Brain **129**(7), 1659–1673 (2006)

[S2] Bartheld, C.S.: Myths and truths about the cellular composition of the human brain: A review of influential concepts. Journal of Chemical Neuroanatomy **93**, 2–15 (2018)

[S3] Trevathan, J.K., Yousefi, A., Park, H.O., Bartoletta, J.J., Ludwig, K.A., Lee, K.H., Lujan, J.L.: Computational modeling of neurotransmitter release evoked by electrical stimulation: nonlinear approaches to predicting stimulation-evoked dopamine release. ACS Chemical Neuroscience **8**(2), 394–410 (2017)

[S4] Arumugasamy, S.K., Chellasamy, G., Gopi, S., Govindaraju, S., Yun, K.: Current advances in the detection of neurotransmitters by nanomaterials: An update. TrAC Trends in Analytical Chemistry **123**, 115766 (2020)

[S5] Fletcher, T.L., De Camilli, P., Banker, G.: Synaptogenesis in hippocampal cultures: evidence indicating that axons and dendrites become competent to form synapses at different stages of neuronal development. Journal of Neuroscience **14**(11), 6695–6706 (1994)

[S6] Caminiti, R., Carducci, F., Piervincenzi, C., Battaglia-Mayer, A., Confalone, G., Visco-Comandini, F., Pantano, P., Innocenti, G.M.: Diameter, length, speed, and conduction delay of callosal axons in macaque monkeys and humans: comparing data from histology and magnetic resonance imaging diffusion tractography. Journal of Neuroscience **33**(36), 14501–14511 (2013)

[S7] Fiala, J.C., Harris, K.M.: Dendrite structure. Dendrites **2**, 1–11 (1999)

[S8] Lewis Jr, T.L., Courchet, J., Polleux, F.: Cellular and molecular mechanisms underlying axon formation, growth, and branching. Journal of Cell Biology

202(6), 837–848 (2013)

[S9] Hawkins, J., Ahmad, S.: Why neurons have thousands of synapses, a theory of sequence memory in neocortex. Frontiers in Neural Circuits **10**, 23 (2016)

[S10] Burkitt, A.N.: A review of the integrate-and-fire neuron model: I. Homogeneous synaptic input. Biological Cybernetics **95**(1), 1–19 (2006)

[S11] De Vries, G.H., Boullerne, A.I.: Glial cell lines: an overview. Neurochemical Research **35**(12), 1978–2000 (2010)

[S12] Ayala, R., Shu, T., Tsai, L.-H.: Trekking across the brain: the journey of neuronal migration. Cell **128**(1), 29–43 (2007)

[S13] Herbert, A.L., Monk, K.R.: Advances in myelinating glial cell development. Current Opinion in Neurobiology **42**, 53–60 (2017)

[S14] Zhang, R., *et al.*: Hybrid deep neural network using transfer learning for EEG motor imagery decoding. Biomedical Signal Processing and Control **63**, 102144 (2021)

[S15] Liu, B., Wang, M., Foroosh, H., Tappen, M., Pensky, M.: Sparse convolutional neural networks. Proceedings of the IEEE conference on Computer Vision and Pattern Recognition, 806–814 (2015)

[S16] Srinivas, S., Subramanya, A., Venkatesh Babu, R.: Training sparse neural networks. Proceedings of the IEEE Conference on Computer Vision and Pattern Recognition Workshops, 138–145 (2017)

[S17] Wong, C., Houlsby, N., Lu, Y., Gesmundo, A.: Transfer learning with neural AutoML. Advances in Neural Information Processing Systems **31** (2018)

[S18] Wen, Y.-W., Peng, S.-H., Ting, C.-K.: Two-stage evolutionary neural architecture search for transfer learning. IEEE Transactions on Evolutionary Computation **25**(5), 928–940 (2021)

[S19] Xue, Y., Wang, Y., Liang, J., Slowik, A.: A self-adaptive mutation neural architecture search algorithm based on blocks. IEEE Computational Intelligence Magazine **16**(3), 67–78 (2021)

[S20] Tan, C., Šarlija, M., Kasabov, N.: Spiking neural networks: Background, recent development and the NeuCube architecture. Neural Processing Letters **52**(2), 1675–1701 (2020)

[S21] D'Ambrosio, D.B., Gauci, J., Stanley, K.O.: HyperNEAT: The first five years. Growing Adaptive Machines, 159–185 (2014)

[S22] Tenstad, A., Haddow, P.C.: DES-HyperNEAT: Towards multiple substrate deep

ANNs. Congress on Evolutionary Computation (CEC), 2195–2202 (2021). IEEE

[S23] Zhou, Y., Yen, G.G., Yi, Z.: A knee-guided evolutionary algorithm for compressing deep neural networks. IEEE Transactions on Cybernetics **51**(3), 1626–1638 (2019)

[S24] Simonyan, K., Zisserman, A.: Very deep convolutional networks for large-scale image recognition. arXiv preprint arXiv:1409.1556 (2014)

[S25] Maimon-Mor, R.O., Makin, T.R.: Is an artificial limb embodied as a hand? Brain decoding in prosthetic limb users. PLOS Biology **18**(6), 3000729 (2020)

[S26] Meng, J., Zhang, S., Bekyo, A., Olsoe, J., Baxter, B., He, B.: Noninvasive electroencephalogram based control of a robotic arm for reach and grasp tasks. Scientific Reports **6**(1), 38565 (2016)

[S27] Tavanaei, A., Ghodrati, M., Kheradpisheh, S.R., Masquelier, T., Maida, A.: Deep learning in spiking neural networks. Neural Networks **111**, 47–63 (2019)

[S28] Barrat, A., Weigt, M.: On the properties of small-world network models. The European Physical Journal B-Condensed Matter and Complex Systems **13**(3), 547–560 (2000)

[S29] An, H., Al-Mamun, M.S., Orlowski, M.K., Yi, Y.: A three-dimensional (3D) memristive spiking neural network (M-SNN) system. 2021 22nd International Symposium on Quality Electronic Design (ISQED), 337–342 (2021). IEEE

[S30] Stanley, K.O.: Compositional pattern producing networks: A novel abstraction of development. Genetic Programming and Evolvable Machines **8**(2), 131–162 (2007)

[S31] Schrum, J., Volz, V., Risi, S.: CPPN2GAN: Combining compositional pattern producing networks and gans for large-scale pattern generation. Proceedings of the 2020 Genetic and Evolutionary Computation Conference, 139–147 (2020)

[S32] Merrild, J., Rasmussen, M.A., Risi, S.: HyperNTM: evolving scalable neural turing machines through HyperNEAT. International Conference on the Applications of Evolutionary Computation, 750–766 (2018). Springer

[S33] Yuen, B., Dong, X., Lu, T.: Supplementary Movie 1 for A 3D Ray Traced Biological Neural Network Learning Model. Nature Communications (2024). Nature Publishing Group
