# Supplementary material for: A 3D ray traced biological neural network learning model
Source: Nat Commun. 2024 Jun 1;15:4693. doi: 10.1038/s41467-024-48747-7 (PMC11525811; doi:10.1038/s41467-024-48747-7)
Supplement: Supplementary file 3 — Description of Additional Supplementary Files [file 41467_2024_48747_MOESM3_ESM.pdf]

Description of additional Supplementary Files

Supplementary Movie 1: RayBNN evolution.
